# Supplementary material for: Unidirectional rotation of micromotors on water powered by pH-controlled disassembly of chiral molecular crystals
Source: Nat Commun. 2023 May 19;14:2869. doi: 10.1038/s41467-023-38308-9 (PMC10198998; doi:10.1038/s41467-023-38308-9)
Supplement: Supplementary file 1 — Supplementary Information [file 41467_2023_38308_MOESM1_ESM.pdf]

Supplementary Information

Unidirectional Rotation of Micromotors on Water Powered by pH-Controlled Disassembly of Chiral Molecular Crystals

Itai Carmeli[1,†], Celine M. Bounioux[1†], Philip Mickel[2†], Mark B. Richardson[3], Yael Templeman[4], Joel M.P. Scofield[5], Greg G. Qiao[5], Brian Ashley Rosen[1], Yelena Yusupov[1], Louisa Meshi[4], Nicolas H. Voelcker[3,6], Oswaldo Diéguez[1,7], Touvia Miloh[8], Petr Král[2,9], Hagai Cohen[10], and Shachar E. Richter[1*]

[1]Department of Materials Science and Engineering, Faculty of Engineering & University Center for Nano Science and Nanotechnology, Tel Aviv University, Tel-Aviv, 69978, Israel.

[2]Department of Chemistry, University of Illinois at Chicago, Chicago, IL 60607, USA.

[3]CSIRO Manufacturing, Bayview Avenue, Clayton, VIC 3168, Australia.

[4]Department of Materials Engineering, Ben-Gurion University of the Negev, Beer-Sheva 84105 POB 653 Israel.

[5]Department of Chemical Engineering, University of Melbourne, Parkville, VIC 3010, Australia.

[6]Drug Delivery, Disposition, and Dynamics, Monash Institute of Pharmaceutical Sciences, Monash University, 381 Royal Parade, Parkville, Victoria, 3052, Australia.

[7]The Raymond and Beverly Sackler Center for Computational Molecular and Materials Science, Tel Aviv University, Tel Aviv 6997801, Israel.

[8]School of Mechanical Engineering, Faculty of Engineering, Tel Aviv University, Tel Aviv University, 69978, Israel.

[9]Department of Physics, Pharmaceutical Sciences, and Chemical Engineering, University of Illinois at Chicago, Chicago, IL 60607, USA.

[10]Department of Chemical Research Support, Weizmann Institute of Science, Rehovot, 76100, Israel.

[†]Equal contribution

*Email: srichter@tauex.tau.ac.il

Supplementary Methods

Materials

Cinchonine >98%, cinchonidine 96%, sodium hypophosphite hydrate, sodium borohydride, ethylene glycol, camphor (R) 99%, purchased from Sigma Aldrich. Silver nitrate (99.9%), purchased from STREM chemicals. Trisodium citrate and sodium decosulfate (SDS) were purchased from Merck.

Methods

Synthesis

Stock solutions of the reaction compounds were prepared: 18 mg of tri-sodium citrate in 12 ml deionized water (DI), 34 mg of sodium hypophosphate in 12 ml DI, 20 mg of sodium dodecyl sulfate (SDS) in 2 ml DI, and 36 mg of silver nitrate in 12 ml DI. 6 mg of cinchonine/cinchonidine were individually partially dissolved in 2 ml DI water containing 100 µL of SDS and stirred for 10 minutes, followed by the addition of 7 ml of DI and to 1 ml of silver nitrate stock solution. The mixture was then stirred for two hours in the dark. Next, 1ml of tri-sodium citrate and 1ml of sodium phosphate stock solutions was drop-wise added to the mixture. The reaction vessel was left to stir for eight consecutive days in the dark until the appearance of chimot aggregates. Then, the solution was centrifuged and washed a couple of times with DI water to recuperate the materials, which were dried for several days under a nitrogen atmosphere. The dry powder was stored in Eppendorf with nitrogen gas for further use.

Experimental setup

A PET glass or Teflon Petri dish (3.5 cm and 7 cm diameter, respectively) was filled with DI water. A single particle was carefully positioned at the air/water interface, and its motion was recorded with a CCD camera mounted on a microscope or by iPhone 6 Plus camera. Analysis of the velocity was done by an image-processing protocol in MATLAB.

UV-Vis measurements were carried out with a CARY 5000 UV-Vis-NIR spectrometer.

Imaging

Optical images were taken using an Olympus BX51 microscope, both with and without a polarization filter. SEM images were done using FESEM or JSM-6300 instruments. TEM investigation was carried out using a 200 kV JEOL JEM 2011, FasTEM, and a JEOL JEM-2100F TEM operating at 200 kV equipped with JED-2300T EDX.

XPS

XPS measurements were performed on a Kratos AXIS-Ultra DLD spectrometer, using a monochromatic Al kα source at a power ranging between 15-75 W and detection pass energies of 20-80 eV. The pressure in the analysis chamber was kept below 10^{-9} torr. Ar-ion sputtering was used only at fine levels, such as to follow the very early stages of removal of adsorbed molecules from the highly corrugated surface. Marked differential charging was typically encountered at the platelet aggregates, which normally complicates the interpretation of XPS results. Here, however, differential charging was exploited to differentiate between signals originated in different domains, which enhances the capabilities of the (large area) XPS probe significantly.[1,2] Samples for the XPS analyses were prepared by carefully lifting Si wafer substrates that were pre-positioned at the bottom of the reaction cell. Thus, platelets floating on the liquid surface could be captured at various stages of the spinning process. To selectively measure the molecules released from the platelets to the water, we removed all observable aggregates from the liquid surface prior to lifting the Si-wafer up. Macroscopic fragments of the Langmuir film could thus be XPS-analyzed. (*1*, *2*)

Surface pressure.

The surface pressure was measured using a Wilhelmy plate attached to a sensor from Nima Technologies. Due to the small difference of surface pressure, pressure was measured using a Petri dish (3.5 cm diameter), at a rate of 2 samples per second. The surface of the water was first cleaned using a vacuum aspirator to remove dust and other possible materials from the surface. Samples of chimots or Camphor particles were measured from the moment they were placed on the surface.

XRD

XRD scans spectra taken by with a Bruker D8 DISCOVER diffractometer using CuKα radiation.

Supplementary Discussion

XPS results

XPS measurements of the platelet aggregates showed the characteristic signals of silver in a non-metallic state, together with the carbon and nitrogen of cinchona alkaloid molecules. Signals from exposed areas of the Si substrate were differentiated by means of controlled surface charging (CSC)(1,2), exploiting the marked difference in electrical conductivity between the platelets and the substrate. We could thus estimate quite accurately the amount of oxygen and carbon at the aggregates, despite the interfering substrate-related signals.

Two peaks in the N 1s line, with roughly equal intensities and a binding energy difference of 2.5-2.8 eV, were found to be a characteristic fingerprint of the bare molecules. Independently, the C/N concentration ratio (after subtraction of related substrate signals) was evaluated, yielding values close to (yet slightly higher than) the theoretical value, which reflects the presence of small amounts of CH-based adsorbents on top of the Ag-cin complex. The aggregate-related oxygen signals yielded a O/N ratio greater than 1, while the bare molecular atomic ratio should be 1. Finally, the Ag 3d line did not exhibit any hint of plasmonic satellites, suggesting that none of the XPS-available regimes can be considered metallic. The latter observation agrees with the elevated charging encountered, as well as with the low Ag atomic ratios, see Supplementary Table 3.

In the following, we describe the main *variations* encountered under different sample treatments and preparation procedures. The N/Ag atomic concentration ratio is given in Supplementary Table 2, providing a measure of the amount of adsorbed molecules. It shows a clear decrease in the amount of platelets after spinning, compared to their amount prior to spinning. Notably, after spinning in BH_4, the amount of cin molecules is even smaller than those detected after spinning in water, in agreement with our direct observations and the faster kinetics achieved upon BH_4 addition to the water.

Analysis of those Si-substrates that were lifted up (after pre-removal of the floating Ag-platelets) provided direct proof for the formation of a well-defined film (believed to be Langmuir- like) by those molecules released during platelets spinning: All expected characteristics of the cinchona alkaloids were found to appear in these films, whereas the amount of silver was practically zero. Similar results were obtained in measurements *near* (off) platelets in the regular samples, yielding vanishing Ag signals.

A fine Ar-sputtering would normally be enough to remove all adsorbed molecules in thin organic coatings. However, such a result was not achieved in the case of chimots. This result is attributed to the rich morphology of the studied particles and the potentially large portion of shadowed areas that could not be sputtered effectively. Yet, based on the very early steps of sputtering (up to 2 min at a flux equivalent to a sputtering rate of ~1A/sec in Ta_2O_5), we could clearly identify the initial removal of cinchona alkaloids (see N/Ag in Supplementary Table 1). Subsequent sputtering steps reached saturation in the N/Ag ratio, suggesting that the removal of cinchona alkaloids in the non-shadowed areas is instantaneous. Additionally, the same behavior was observed *after* spinning, indicating that even after the spinning was terminated, many molecules remained in the Ag aggregate. This result agrees with our experiments demonstrating that the chimots resume their spinning motion upon replacement of the solvent with fresh water.

Detailed features are indicated by Supplementary Table 2:

1. The **N/Ag concentration ratio**:
    a. Before spinning – the same value is obtained for cin+ and cin-, in agreement with expectations.
    b. After spinning in water (until the motion was terminated for the first time) - N/Ag decreases, indicating the release of molecules during spinning, but also that the release is not complete.
    c. Bottom side of the plates – N/Ag gives even lower values, suggesting that the release takes place at the water-platelet interface. Note that most of our samples consisted of a random combination of top vs bottom faces, in contrast to this dedicated experiment.

d. After spinning in BH_4 – a larger decrease is observed in N/Ag when compared to spinning in water, which correlates well with the direct observations of quicker spinning in BH_4. Note that the decrease is more moderate for cin- compared to cin+. Together with point b above, it may reflect a lower release-efficiency for cin-. However, the statistical validity of this conclusion cannot be stated confidently due to the lack of data points for cin-, which were all taken from the same propelling experiment.

e. After sputtering – N/Ag decreases, as expected from molecules coating a silver-containing substrate/core. Two additional comments should be noted in this respect:

i. Saturation was quickly reached, such that continued sputtering did not quite affect it. The reason for that is that the surfaces of the samples are very rough, with at least 50% of the area shadowed from the ion beam. Such morphology offers huge *surface area available for the spinning mechanism*, a feature supported by other findings as well.

ii. The value of N/Ag before sputtering is too large to be associated with a thin layer coating on a flat surface. Extensive roughness needs to be considered, with multilayers of molecules residing within the shadowed portions. Alternatively, hybrid arrangements with nm-sized cores of Ag surrounded by cinchona alkaloids can account for the observed N/Ag ratios.

f. Langmuir film – Very high N/Ag values were obtained with large variations between different points, reflecting the very low Ag signals in these samples. The table presents a selected single value corresponding to a more reliable data point for the derivation of O/N (see experimental section). The N/Ag values indicate that the inspected film is not totally free of particles. Most of the particles were indeed removed prior to lifting the substrate up, but residual small particles remained (small enough to have no significant effect on the other molecule-related values discussed here).

2. **The C/N concentration ratio**:

a. Before spinning – Similar values are obtained for cin- and cin+; both are high when compared to the theoretical value of 9.5. We associate this result with

contaminants, such as extra hydrocarbon molecules that are likely situated on top of the cin± molecules.

b. After spinning in water – the C/N ratio increases. This is consistent with a mechanism where the cin+ and cin- molecules are *released as a whole,* while potential hydrocarbon contaminants are not as readily released into the water. This conclusion is further confirmed by the ‘bottom’ sides, which more readily release molecules; hence the C/N ratio is found to be even higher.
c. After sputtering – A small decrease is obtained, as expected from the removal of the entire molecule. Note that sputtering of the C-H moieties should give rise to more pronounced changes in the ratio of total C/N.
d. After spinning in BH_4 – For cin-, the change is consistent with point 2b above and is indeed even higher, in agreement with the N/Ag data.
e. Langmuir film – Deviations from the theoretical expectation similar to the above were obtained, however, with a slightly lower amount of extra carbon.

3. **The O/N concentration ratio** –

The amount of O provided is after subtracting the SiO_2 signal. In general, the values vary between 1 and 2, while the theoretical value for the bare molecule is O/N=1. Results for the various samples are as follows:

a. Before spinning – there is a slight extra O for both cin+ and cin-. Extra O can arise from adsorbed molecules containing water or OH groups. Additionally, extra O can arise from additional O-atoms involved in the binding to the silver.
b. After spinning in water – A clear increase in the O/N ratio is found for both cin+ and cin-. This increase may suggest a process where the release of molecules involves replacement by O, such as replacement by water molecules.
c. After sputtering – A clear decrease in the O/N ratio is seen both before and after spinning in the water, which suggests the presence of extra O-containing molecules (like water or C-ox).
d. After spinning in $NaBH_4$ – Reliable values were unable to be attained due to uncertainties as with the differential charging analysis (the CSC).

e. <u>Langmuir film</u> – Interestingly, data in these cases gave binary values: either O/N=1 or 2.

4. **N1/N2**: For cases with a high release (low N/Ag) we find increased N-reduction vs N-oxide (details not provided in the table).
5. **Ag Auger** - The Auger line of silver (not included in the table) did give indications for an increased metallic component after spinning, especially in the $NaBH_4$ solvent.

Simulation

In the absence of noise, one may use the following generalized Stokes' law for modeling the hydrodynamics of chimots. The effective force ***(F)*** and torque ***(T)*** acting on the particle are related to its translational velocity $(\boldsymbol{v})$ and angular velocity ***(ω)*** via the grand-resistance (dissipation) matrix ***(H)***[3]: $\begin{pmatrix}\boldsymbol{F}\\ \boldsymbol{T}\end{pmatrix} = \eta\, \boldsymbol{H} \begin{pmatrix}\boldsymbol{v}\\ \boldsymbol{\omega}\end{pmatrix}$, where η denotes the dynamic viscosity of the fluid. The above formulation holds, providing the characteristic Reynolds number is small. The Reynolds number is given by: $Re = \rho v L/\eta$, where L is a typical linear dimension of the chimot and ρ *is the* fluid density. Typical values for water at room temperature are $\rho \approx 1000$ kg/m^3 and $\eta \approx 1$ cP. The chimot on the left of Figure 3f has a cross-section of 0.33 mm^2, which corresponds to an equivalent disk with radius L = 0.32 mm; together with a speed v ≈ 0.73 mm/s, this leads to Re ≈ 0.2. The other chimot in Figure 3f has a cross-section of 1.21 mm^2 and v ≈ 1.71 mm/s, resulting in Re ≈ 1.

We employ here a two-dimensional self-propulsion model, similar to that reported by Kuemmel[4] for studying the motion of micron-sized L-shaped particles made of light-sensitive material. In this case, one gets:

$$\begin{pmatrix} Fcos(\varphi) \\ Fsin(\varphi) \\ T \end{pmatrix} = \begin{pmatrix} H_{xx} & 0 & H_{x\varphi} \\ 0 & H_{xx} & H_{x\varphi} \\ H_{x\varphi} & H_{x\varphi} & H_{\varphi\varphi} \end{pmatrix} \begin{pmatrix} vcos(\varphi + \theta \\ vsin(\varphi + \theta \\ \dot{\varphi} \end{pmatrix} \qquad [2]$$

where φ describes the orientation of the force in the lab's frame of reference and θ represents the angle between the force and the translational velocity v. To simplify the formulation, we enforce the following symmetries in the grand-resistance matrix, namely: H_{yy}=H_{xx}, H_{xy}=H_{yx}=0,

$H_{x\varphi}$=$H_{\varphi x}$=$H_{y\varphi}$=$H_{\varphi y}$. In turn, the corresponding parameters in the grand-resistance matrix can be accordingly expressed in terms of a typical linear size r of the particle as: $H_{xx} = \beta_{xx} r$, $H_{x\varphi} = \beta_{x\varphi} r^2$, and $H_{\varphi\varphi} = \beta_{\varphi\varphi} r^3$. Note that for a freely suspended spherical particle of radius r in the Stokes regime, $\beta_{xx} = 6\pi$, $\beta_{x\varphi} = 0$ and $\beta_{\varphi\varphi} = 8\pi$, but for our geometries, these factors take other numerical values. In this study, we select r as the radius of an equivalent circle with an area equal to that of the parallel projection of the particle on a plane above it and parallel to the water surface. For the first particle, we get r = 0.32 mm, and for the second particle, r = 0.62 mm. The resulting equations render an analytical solution given by:

$$\tan\frac{\varphi}{2} = \frac{\beta_{x\varphi}\, F\, r}{\beta_{x\varphi}\, F\, r + \beta_{xx}\, T}\left(1 - \sqrt{\left(\frac{\beta_{xx}}{\beta_{x\varphi}}\,\frac{T}{F\, r}\right)^2 - 2}\;\tan\left\{\frac{\sqrt{\beta_{xx}^2\, T^2 - 2\beta_{x\varphi}^2 F^2 r^2}}{4\,\beta_{x\varphi}^2 - 2\beta_{xx}\beta_{\varphi\varphi}}\,\frac{t}{\eta\, r^3}\right\}\right), \tag{2}$$

which is subjected to the initial condition, $\tan\frac{\varphi(0)}{2} = \frac{\beta_{x\varphi}\, F\, r}{\beta_{x\varphi}\, F\, r + \beta_{xx}\, T}$.

Once φ is known, the speed v can be readily computed as:

$$v^2 = \frac{F^2}{\eta^2 \beta_{xx}^2\, r^2} - \frac{2T}{\eta\, \beta_{xx}\, r}\dot{\varphi} + 2\, r^2\left(\frac{\beta_{\varphi\varphi}}{\beta_{xx}} - \frac{\beta_{x\varphi}^2}{\beta_{xx}^2}\right)\dot{\varphi}^2, \tag{3}$$

and the angle of the velocity vector can be directly determined from $\tan(\varphi + \theta) = \frac{F \sin\varphi - \eta\beta_{x\varphi}\, r^2 \dot{\varphi}}{F\cos\varphi - \eta\beta_{x\varphi}\, r^2\dot{\varphi}}$.

It is not easy to analytically determine the behavior of φ, v, and θ from these equations, but an asymptotic analysis in terms of a small parameter $\beta_{x\varphi}/\beta_{xx}$, renders (to leading- order) the following expressions:

$$\varphi \approx \frac{T}{\eta\,\beta_{\varphi\varphi}\, r^3}t + \frac{\beta_{x\varphi}}{\beta_{xx}}\frac{Fr}{T}\left(1 + \cos\frac{T}{\eta\,\beta_{\varphi\varphi}\, r^3}t - \sin\frac{T}{\eta\,\beta_{\varphi\varphi}\, r^3}t\right), \tag{4}$$

$$v \approx \frac{F}{\eta\,\beta_{xx}\, r} - \frac{\beta_{x\varphi}}{\beta_{xx}}\frac{T}{\eta\,\beta_{\varphi\varphi}\, r^2}\left(\cos\frac{T}{\eta\,\beta_{\varphi\varphi}\, r^3}t + \sin\frac{T}{\eta\,\beta_{\varphi\varphi}\, r^3}t\right), \tag{5}$$

$$\theta \approx \frac{\beta_{x\varphi}}{\beta_{xx}}\frac{\beta_{xx}}{\beta_{\varphi\,\varphi}}\frac{T}{Fr}\left(\sin\frac{T}{\eta\,\beta_{\varphi\varphi}\,r^3}t - \cos\frac{T}{\eta\,\beta_{\varphi\varphi}\,r^3}t\right). \tag{6}$$

The expansion of the linear velocity provides a simple way for fitting the experimental results of Fig. 3(f) to our model. The period τ in the experimental results can be identified as $\tau = \eta\beta_{\varphi\varphi}r^3 2\pi/T$, its average velocity $\bar{v} = F/(\eta\beta_{xx}r)$, and the difference between its maximum and minimum by $\Delta v = 2\sqrt{2}(\beta_{x\varphi}/\beta_{xx})\ (T/\eta\beta_{\varphi\varphi}r^2)$. In other words, given the experimental results for η, r, τ, $\bar{v}$, and Δv, we can specify the parameters governing our model as $T/\beta_{\varphi\varphi} = 2\pi\eta r^3/\tau$, $F/\beta_{xx} = \eta r\bar{v}$, and $\beta_{x\varphi}/\beta_{xx} = \Delta v\tau/(4\pi\sqrt{2}r)$. The quotient $\beta_{xx}/\beta_{\varphi\varphi}$ affects the distance traveled by the particle during one period (τ) and is adjusted here to fit the experimental results.

Using this scheme, it is possible to explicitly determine the values of F/β_{xx}, $T/\beta_{\varphi\varphi}$, $\beta_{x\varphi}/\beta_{xx}$, and $\beta_{xx}/\beta_{\varphi\varphi}$ resulting in the numerical simulations depicted in Fig. 3f (including a comparison with experiments), which include the trajectories obtained from numerically integrating the exact velocity expression. For the first chimot, we find F/β_{xx} = 0.24 nN, $T/\beta_{\varphi\varphi}$ = −0.039 nN·mm, $\beta_{x\varphi}/\beta_{xx}$ = 0.11, and $\beta_{xx}/\beta_{\varphi\varphi}$ = 0.24. For the second chimot, we find F/β_{xx} = 1.08 nN, $T/\beta_{\varphi\varphi}$ = 0.39 nN·mm, $\beta_{x\varphi}/\beta_{xx}$ = 0.41, and $\beta_{xx}/\beta_{\varphi\varphi}$ = 0.077. Realizing that some noise may still exist in the system, for example due to Brownian motion or the irregular solvation of the chimots, the experimental trajectories slightly differ from the computed ones, as seen especially in the case of the second chimot of Fig. 3f.

Although the above simplified hydrodynamic model doesn't capture the microscopic mobility mechanisms of the chimots, it does provide a useful insight into the kinematical behavior of the chimots. To begin with, it shows that the motion of chimots is compatible with standard propulsion mechanisms, where the acting force is always oriented in the same direction with respect to a body-fixed coordinate system (such as the gradient force due to surface tension under uneven solvation of the chimot). Assuming that the geometrical factors β_{xx} and $\beta_{\varphi\varphi}$ are of the same order of magnitude as those for a perfect sphere, we can deduce that the hydrodynamic forces and torques exerted on the chimots are of the order of a few nN and nN/mm, respectively. The proposed model also helps us to explain the fact that there is no clear correlation was observed in the experiments between the speed and size of the chimots. The force depends linearly on r, while the torque depends quadratically on r, therefore canceling any dependence of the speed on the size, if second-order terms are neglected.

We could try to use the MD simulation results to provide a rough estimate of the driving forces acting on a single chimot to justify the forces used in the above hydrodynamic model. The calculated energies presented in Fig. 3b show that the (-1 0 0) and (1 0 0) charged facets release molecules to the surrounding water, with a slight difference in their release rates (roughly 10%). For simplicity, consider that the surface tension of water drops to 50% of its original value of γ_{facet} = 36 nN/μm (γ_{water} = 72 nN/μm) when fully saturated with amphiphilic cin molecules. We can also assume that the surface coverage above a particular facet is roughly proportional to its ejection rate of the cin molecules. This rate is proportional to the difference in binding energies to water and the crystal, ΔE_{facet}. Consequently, the surface tension above a particular facet can be estimated from $\gamma_{facet} = \gamma_{water} - c\cdot(\gamma_{water} - \gamma_{saturated})\cdot\Delta E_{facet}$, where c accounts for the diffusion (removal) of released molecules. If the transport of released molecules is fast, but there is still a 10% difference in release rates between the facets, we can estimate the differences in surface tension across the crystal to $\Delta\gamma_{crystal}$ = 3.6 nN/μm. From $\Delta E_{facet} = \gamma_{facet}\cdot L\cdot\Delta x$, we can estimate the energy change of a liquid surface of γ_{facet} that is in contact with the facet of a length L and that moves in the orthogonal direction by Δx. The force acting on this facet, $F_{facet} = \Delta E_{facet} / \Delta x = L . \gamma_{facet}$, produces a total force of $\Delta F_{crystal} = L\cdot \Delta\gamma_{crystal}$ = 9 nN, which is acting on a crystal with the side length of L = 2.5 μm, that is exposed to the surface tension difference of $\Delta\gamma_{crystal}$ = 3.6 nN/μm. A chimot with a radius of r=1 mm can accommodate N~1,250 of such crystals with adequate separations, giving a torque of $\tau = N . \Delta F_{crystal} . r$ = 11 μN·mm. These values are about 3 orders of magnitude larger than our hydrodynamical estimates for the forces (4.5 nN and 18 nN) and torques (1 nN.mm and 10 nN.mm) acting on the two particles (crystals) of radius (0.32mm and 0.62mm) used in our Stokes simulations, resulting in the trajectories depicted in Fig. 3(d). The large difference between these forces can be related to the much smaller difference in the concentration of the molecules around the crystallites (constant c), a smaller asymmetry in crystallites positioned at the chimot surface (different orientations and submergences), and the complex shapes of chimots, further decreasing the number of active crystallites. At the same time, one can expect that when these parameters optimized one can significantly increase the driving of such systems.

Supplementary Figures

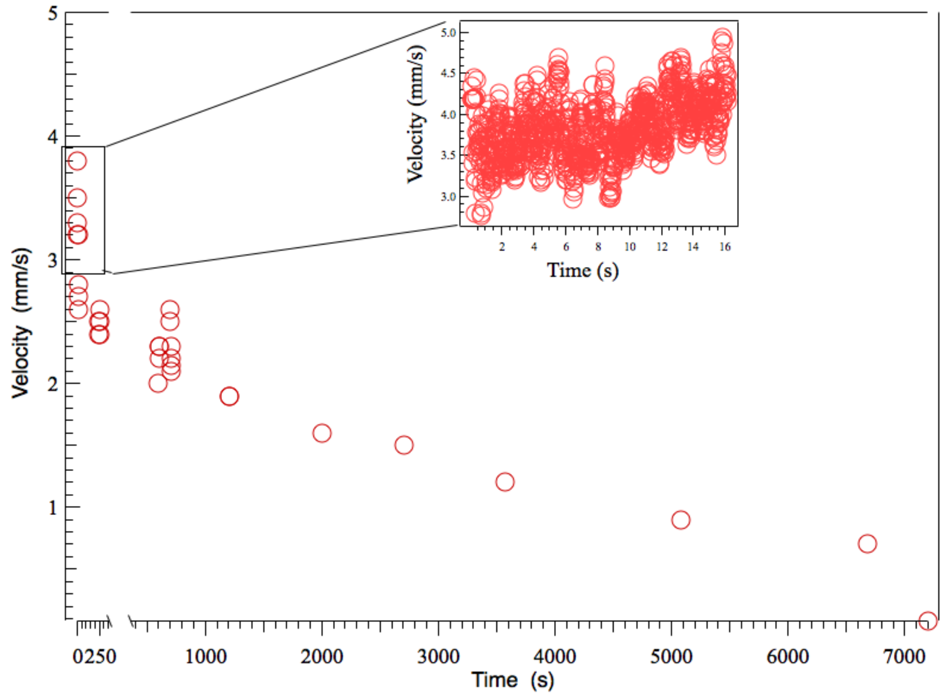

Supplementary Figure 1. Three hours recording of chimots' velocities. Most chimots terminate their motion within 20 seconds. Few chimots continue rotating between 1000-7000 seconds.

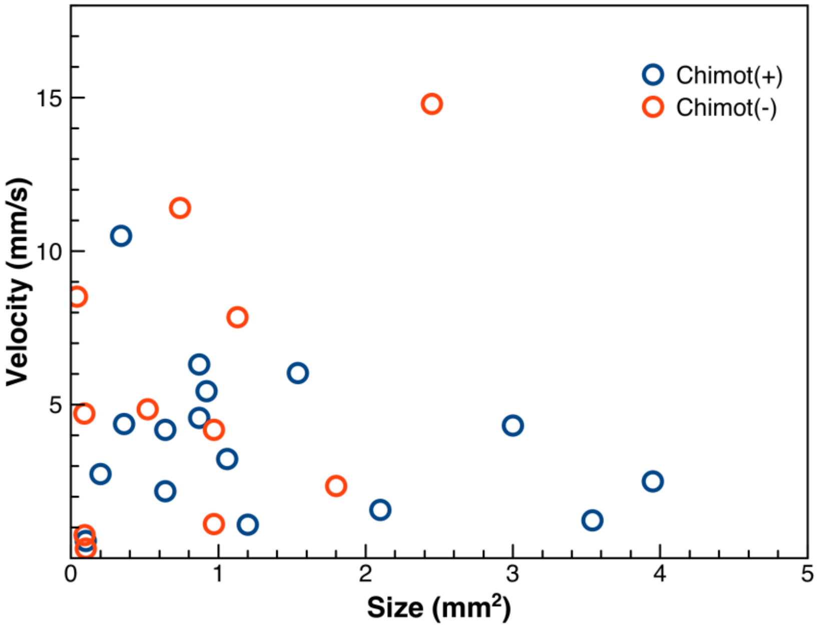

Supplementary Figure 2. Size *vs* velocity of chimots. The velocity of the chimots tends to be greater for smaller particles. As it is expected that the chimots are propelled by ejected surface molecules, smaller particles have larger surface area to volume (and therefore mass) ratios, leading to larger velocities.

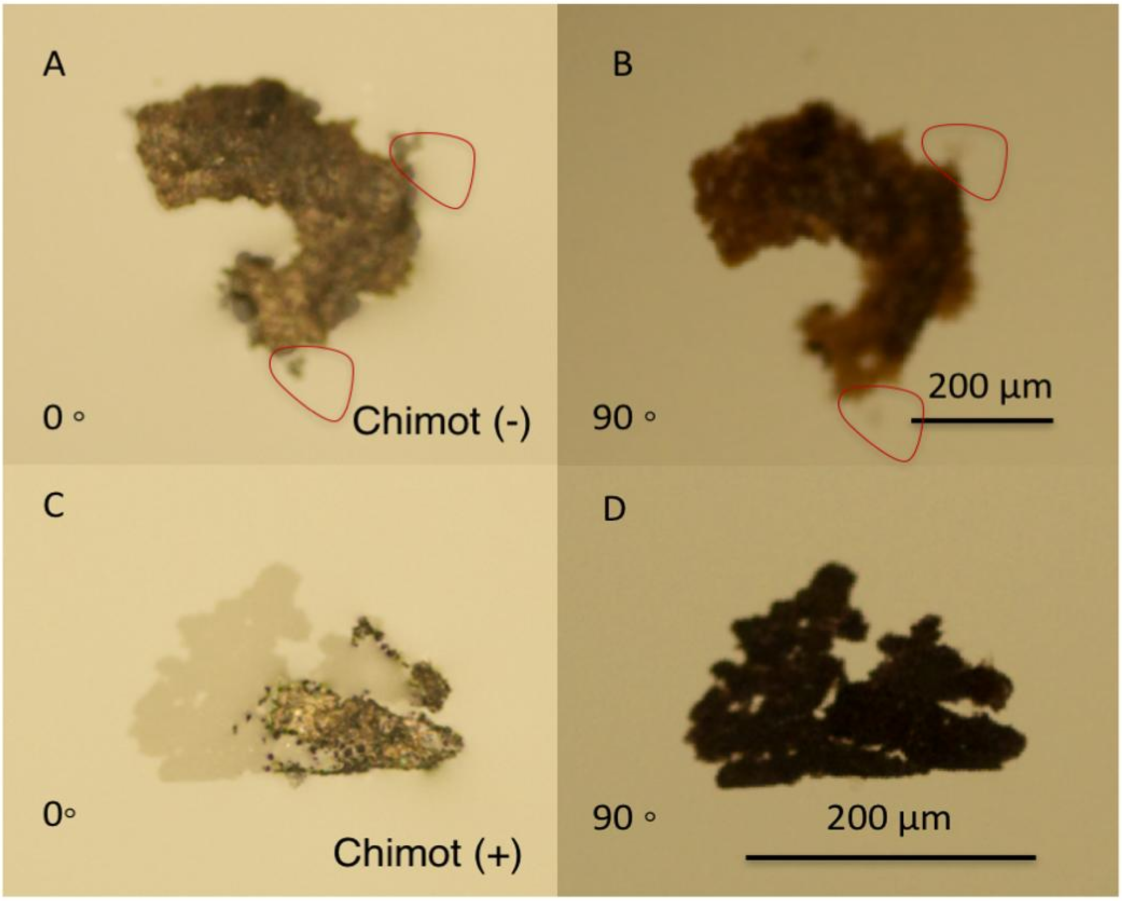

Supplementary Figure 3. Polarized Microscopy images. A. Chimot (-) with polariser at 0°. B. Polariser at 90°. C. Chimot (+) with polariser at 0°. D. Polariser at 90°. In Supplementary Fig 5 we observe partial left and right polarization of light. The chimot changes in brightness when the polarizer is rotated.(*5*)

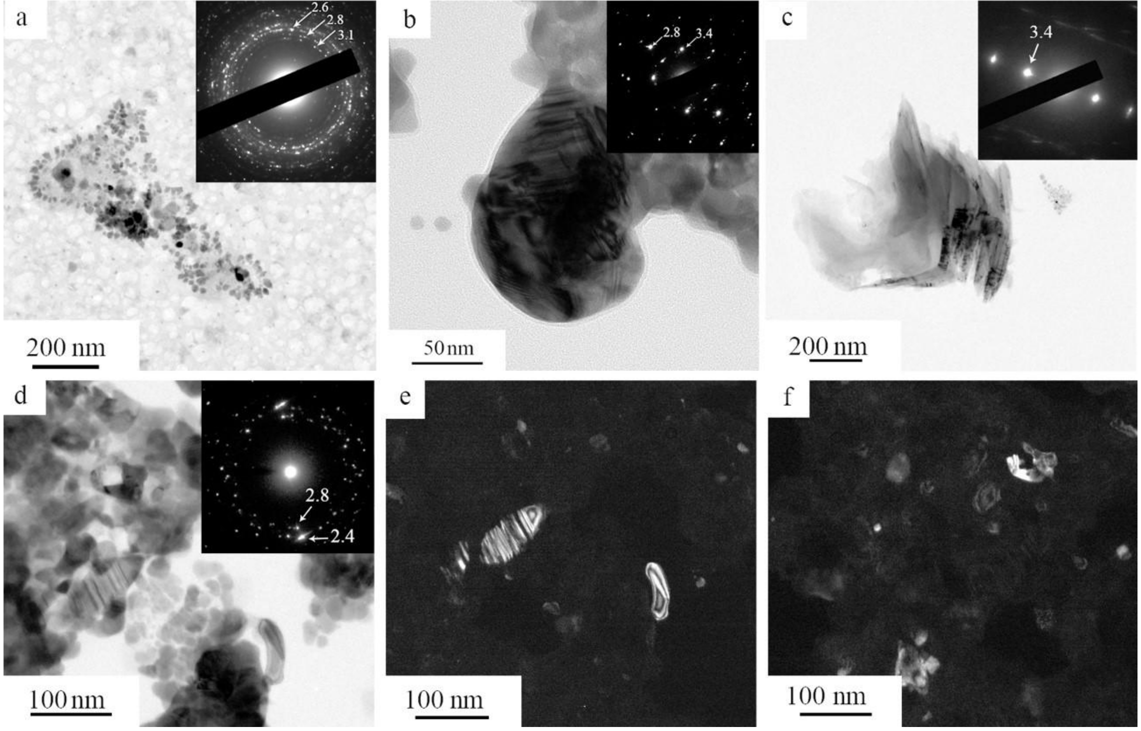

Supplementary Figure 4. (a)-(c) TEM images and electron diffraction patterns (in the insets) taken from the chimot (+; (d)-(f) TEM images and electron diffraction patterns taken from the chimot (-).
Chimot (+) clusters contain several populations of particles, exhibiting different sizes and morphologies. Fig (a) exhibits a general view of one typical cluster and its electron diffraction pattern in the inset. On this pattern, d_{hkl} values (in Å) of three first rings are shown. D_{hkl}s of pure Ag do not appear on any electron diffraction patterns taken from this material. Figs (b) and (c) show particles with high density of planar defects and layered particles, respectively. Their electron diffraction patterns are shown in the insets with largest d_{hkl}s values marked, showing that these particles do not exhibit Ag structure, although using EDS analysis Ag was measured in these particles.
Fig (d) shows typical cluster of the chimot (-) material. Corresponding electron diffraction pattern is shown in the inset. D_{hkl} value of 2.4 Å (corresponding to the most intense first peak of Ag structure) is marked. Other d_{hkl} (corresponding to different structure) is also marked. Fig (e) and (f) are dark field TEM images taken in g=2.4 and 2.8 Å, respectively, lighting up Ag particles with typical twin/stacking faults in fig (e) and particles with different structure in fig (f).

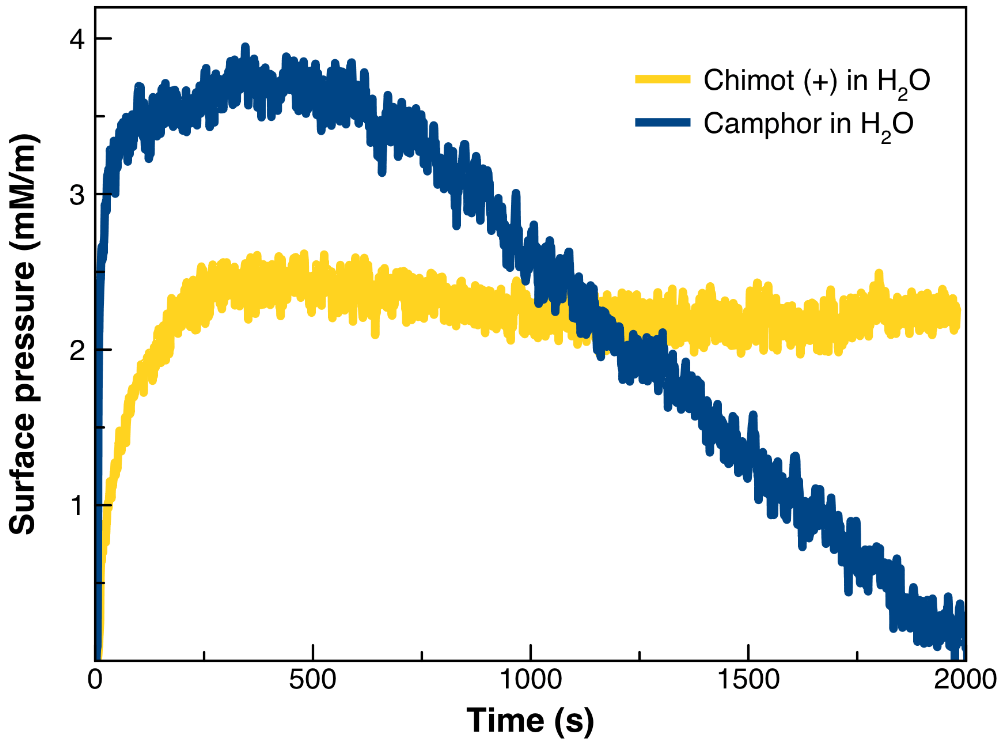

Supplementary Figure 5. Surface pressure isotherms of camphor (-) and chimot (+) particles in H_2O. The surface pressure of the motors remains constant after initially rising to its equilibrium surface pressure, while the surface pressure of the camphor initially shows a maximum surface pressure before decreasing due to the evaporation of molecule that surface.

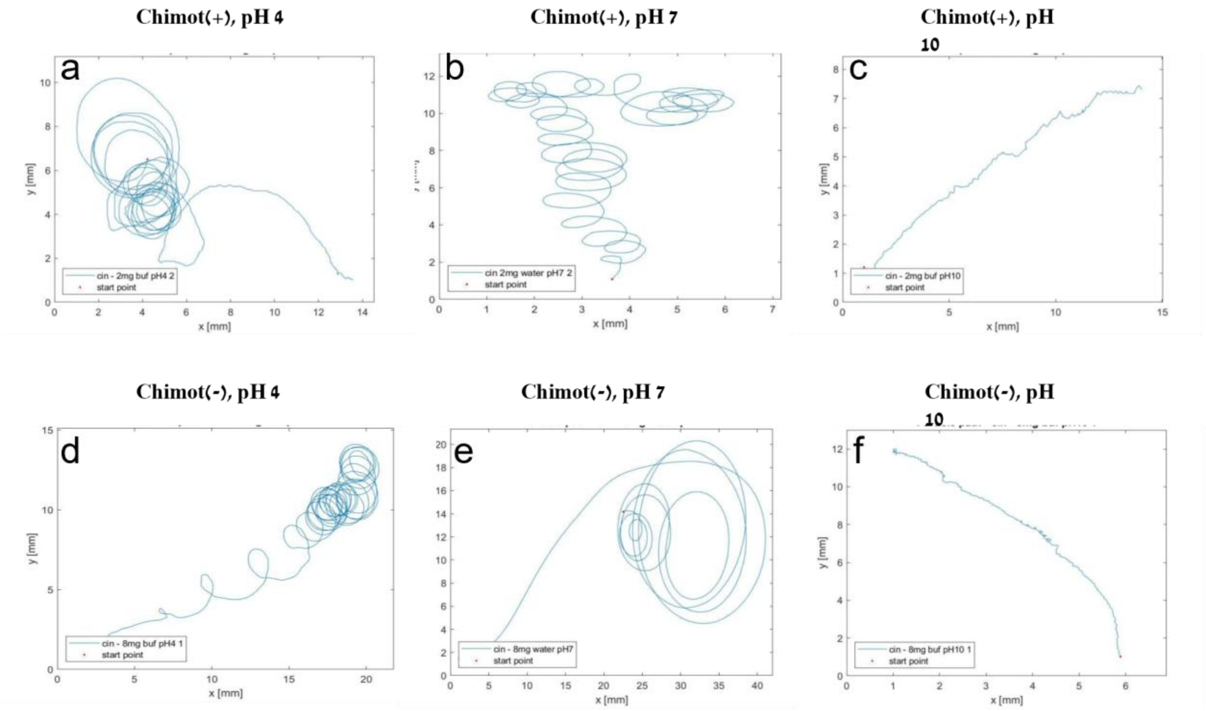

Supplementary Figure 6. Effect of pH on chimot path. (a), (b), (c) represent center of mass coordinates of chimot(+) at pH 4, 7, 10 respectively; (d), (e), (f) represent center of mass coordinates of chimot(-) at pH 4, 7, 10 respectively. The chimots follow a circular path on the surface in neutral or acidic environments. In basic environments, the chimots no longer follow circular paths.

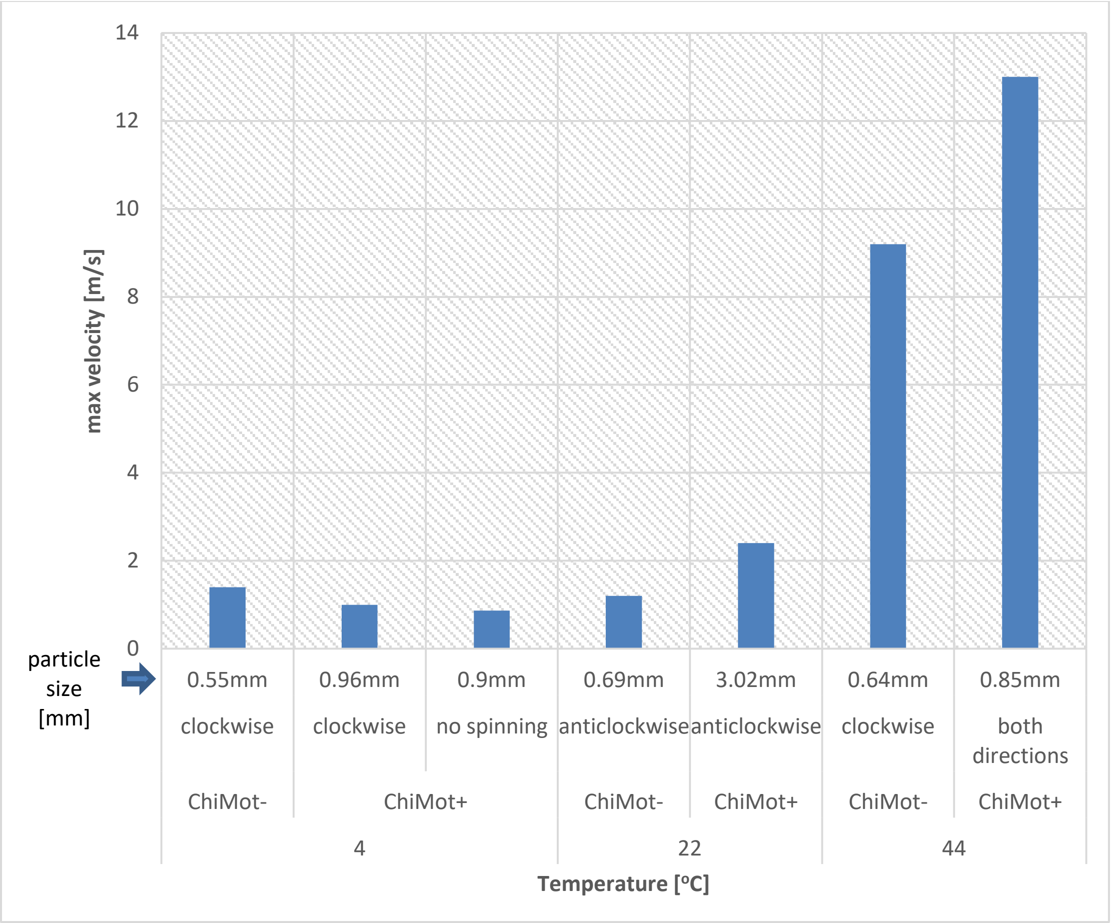

Supplementary Figure 7. Dependency of chimot maximum linear velocity on water temperature. Dependency of chimot maximum linear velocity on water temperature, pivot chart. Axis X represents several levels of grouping. Bottom row of axis X represents temperature of the water; next chimot type that was tested at each temperature; above it, direction of rotation; and upper level shows initial particle size of tested particle. Maximum linear velocity for each particle is represented by blue columns. The temperature dependence of the chimots' rotation is consistent with the proposed mechanism in that the kinetics of protonation and ejection of surface molecules will increase with higher temperatures.

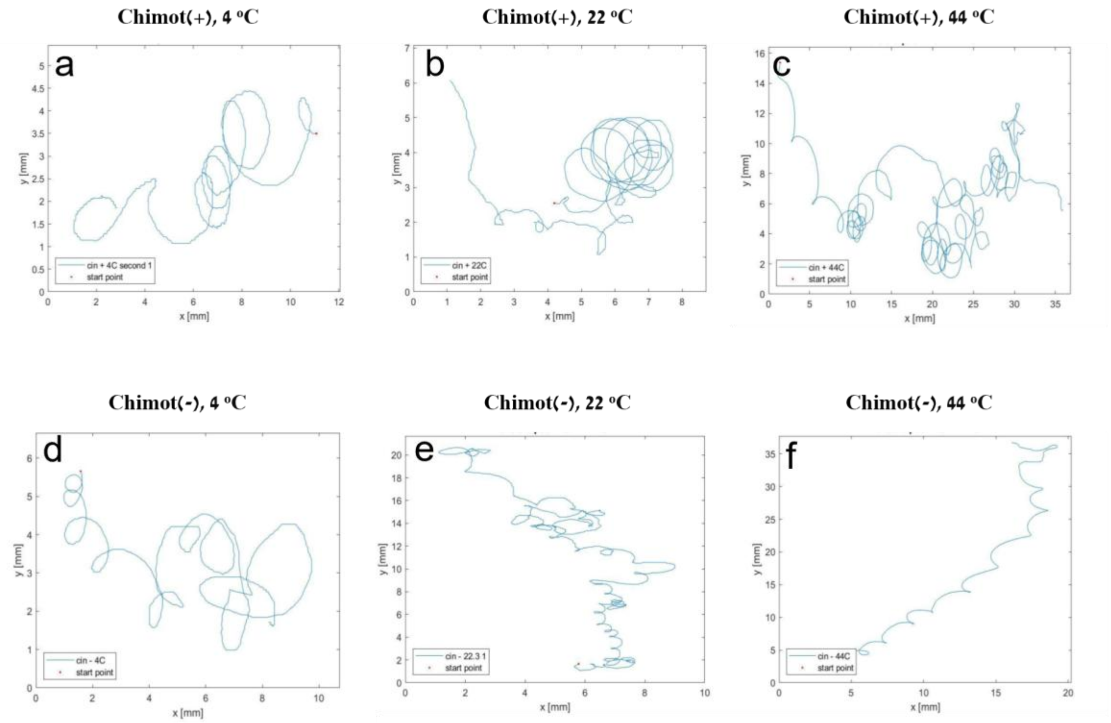

Supplementary Figure 8. Chimots path at 4 °C, 22 °C and 44 °C. (a-c) center of mass coordinates of chimot(+) at temperatures 4, 22 and 44 °C respectively; (e-f) Center of mass coordinates of chimot(-) at temperatures 4, 22 and 44 °C respectively.

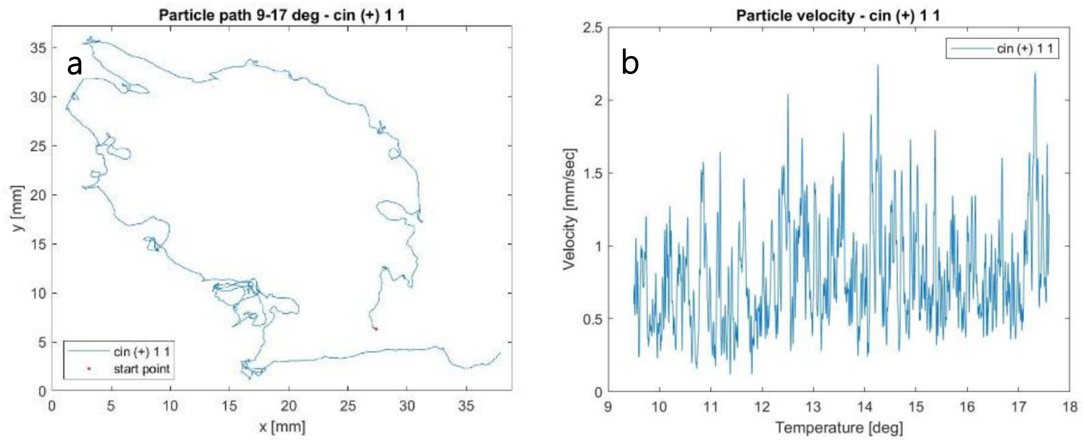

Supplementary Figure 9. 1st trial, chimot particle #1, path **(a)** and linear velocity **(b)** from 9 to 17.5 °C.

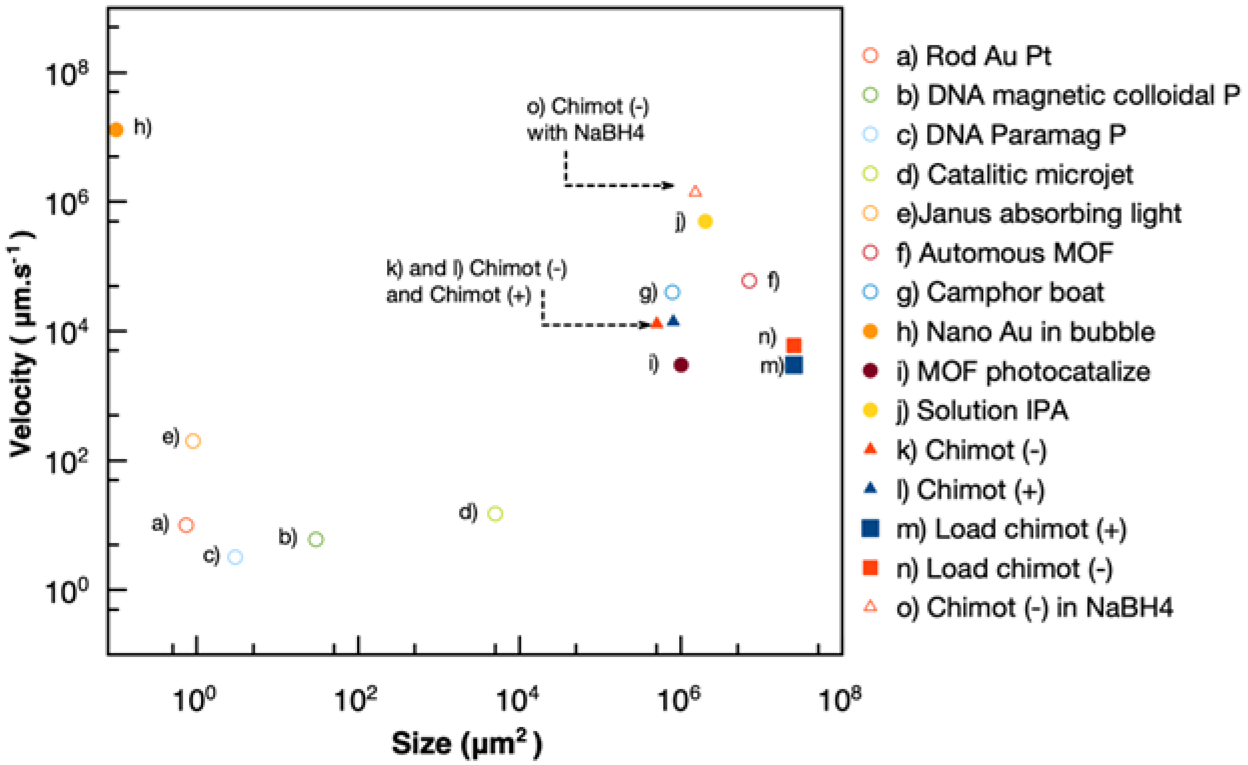

Supplementary Figure 10. Comparison of chimots' speeds to other artificial and biological swimmers Chimots accelerated with $NaBH_4$ exhibit velocities exceeding that of any prior reports. See Supplementary Table 3.

Supplementary Tables

Batch	Chimot	clockwise	anticlockwise	no rotations	total
1	Chimot(+)	5	22	3	30
2	Chimot(+)	12	20	10	42
3	Chimot(+)	10	25	10	45
4	Chimot(+)	3	12	0	15
5	Chimot(+)	2	11	1	14
6	Chimot(-)	13	5	13	31
7	Chimot(-)	17	5	6	28
8	Chimot(-)	7	3	3	13
9	Chimot(-)	5	1	0	6
10	Chimot(-)	6	2	1	9

Supplementary Table 1. Representative data of 10 batches.

Batch	Chimot	clockwise	anticlockwise
1	Chimot(+)	19%	81%
2	Chimot(+)	38%	63%
3	Chimot(+)	29%	71%
4	Chimot(+)	20%	80%
5	Chimot(+)	15%	85%
6	Chimot(-)	72%	28%
7	Chimot(-)	77%	23%
8	Chimot(-)	70%	30%
9	Chimot(-)	83%	17%
10	Chimot(-)	75%	25%

Supplementary Table 2. Representative particle statistics. Rotation direction tends to be anticlockwise for chimot(+) and clockwise for chimot(-). This suggests that enantiomers should experience opposite rotation directions.

			C/N*	N/Ag	O/N*
**Cin^{+}/Ag**	**before**	Initial<br><br>Sputt.	16.0<br><br>14.7	3.0±0.05<br><br>2.15	1.22<br><br>0.77
	**after H_2O**	Initial<br><br>Sputt.	17.3<br><br>24.6	2.5±0.05<br><br>1.25---1.07	1.72<br><br>1.2
		Bottom	19.0	1.65±0.1	
		Langmuir	14.5	33.6±1	1.0 or 2.0 ±0.1
	**after $NaBH_4$**		14.6	1.1±0.1	
**Cin^{-} /Ag**	**before**		16.1<br>14.1	3.05±0.1	1.1±0.15
	**after H_2O**		21.3	3.0±0.1	~2
	**after $NaBH_4$**		25±4	1.95±0.1	

Supplementary Table 3. XPS data recorded from samples before spinning and after spinning in water or BH4. Selected atomic concentration ratios (C/N, O/N and N/Ag) are given, corresponding to the aggregate-related components only. Data after moderate sputtering are provided for two representative cases. The label 'Bottom' stands for samples where Ag aggregates were carefully turned up-side-down, such as to selectively measure the face that was (during spinning) directly exposed to the water. Langmuir stands for samples lifted after removal of the Ag platelets from the water surface, such as to selectively measure the Langmuir film of molecules released from the platelets. After placement onto the surface of water, organic molecules appear to be lost from the chimot to the surrounding water. However, silver does not appear to be ejected from the chimots. Addition of NaBH4 results in faster loss of organic molecules from the chimots.

* Aggregate-related components only

**Microswimmer**	**Propulsion Mechanism**	**Medium**	**Size (μm^2)**	**Max speed ($\mu m. s^{-1}$)**	
Janus spherical particles with acatalytic Pt patch (*7*)	Self-diffusiophoresis catalyzed by a chemical reaction on the Pt surface	H_2O_2 aqueous solution	1.6	3	a
DNA-linked anisotropic doublets composed of paramagnetic colloidal particles (*8*)	Rotation induced by a rotating magnetic field	Near a boundary in aqueous solution	3	3.2	b
Chiral colloidal propellers(*9*)	External actuation by a magnetic field	Aqueous solution	0.5	40	c
Janus particles half-coated with Au	Self-thermophoresis due to local heating at the Au cap	Aqueous solution	1	10	d
Catalytic microjets(*10*)	H_2O_2 catalysis on the internal surface of the microjet	H_2O_2 aqueous solution	50	10^4	e
Nano Gold optical beam(16)	Nano Gold	Buble cavity	0.1	$1.7x10^7$	f
MOF photocatalytic micromotor (15)	Photo catalytic bubble	H_2O_2	$1x10^6$	3000	i
IPA bubble on water (14)	Marangoni flow	Aqueous medium	$3 x10^6$	$0.5x10^6$	j
Autonomous Motor Framework Peptide (*12*)	Marangoni flow	Aqueous medium	$0.78x10^6$	$4x10^4$	k
Camphor boat(*13*)	Marangoni flow	Aqueous medium	$7 x10^6$	$6x 10^4$	l
Chimot(-)	Marangoni flow	Aqueous medium	$0.1- 5x 10^6$	$0.2 - 1.3x10^4$	m
Chimot (+)	Marangoni flow	Aqueous medium	$0.2 – 50x 10^6$	$0.2 -1.4x10^4$	n
Chimot (+) load	Marangoni flow	Aqueous medium	$2.5 x10^8$	$4x 10^3$	o
Chimot(-) load	Marangoni flow	Aqueous medium	$2.5 x10^8$	$6x10^3$	p
Chimot(+) $NaBH_4$	Marangoni flow	Aqueous medium $NaHB_4$	10^6	$1.4 x10^6$	q

Supplementary Table 4. Artificial swimmers- velocities .

ChiMot	Temp [^{o}C]	clockwise	anticlockwise	anticlockwise/clockwise	no rotation
cin(-)	4.5	23%			77%
	23	46%	8%		46%
	44	45%	45%		10%
cin(+)	4.5	10%	20%		70%
	23	70%	20%		10%
	44	44%	44%	11%	0

Supplementary Table 5 . Effect of water temperature on chimot motion. For both chimot- and chimot+ at low temperature of 4.5^{o}C most particles did not rotate. The number of rotating particles increases with the temperature.

Trial	Particle #	T_{start} [^{o}C]	$T_{rotations}$ [^{o}C]	Max vel [mm/s]	Direction	@ min:s
1	1	9	No	NR	NR	NR

			rotations			
	2	11.5	11.5	11	clockwise	0:00
			23.5	11	clockwise	8:20
			32.5	10	clockwise	13:00
			33	10.5	clockwise	14:00
2	1	8	No rotations	NR	NR	NR
	2	10	24-26	6.2	anticlockwise	8:30
	3	24	24-26	23	clockwise	0:00

Supplementary Table 6. Effect of temperature on chimot motion (continuous experiment).

Supplementary References

1. K. Shabtai, I. Rubinstein, S. R. Cohen, H. Cohen, High-Resolution Lateral Differentiation Using a Macroscopic Probe: XPS of Organic Monolayers on Composite Au−SiO2 Surfaces. *J. Am. Chem. Soc.* **122**, 4959–4962 (2000).

2. I. Doron-Mor *et al.*, Controlled surface charging as a depth-profiling probe for mesoscopic layers. *Nature*. **406**, 382–385 (2000).

3. F. Kuemmel *et al.*, Circular Motion of Asymmetric Self-Propelling Particles. *Phys. Rev. Lett.* **110,** 198302 (2013).

4. Hagen, B. ten *et al.* Can the self-propulsion of anisotropic microswimmers be described by using forces and torques? *J. Phys.: Condens. Matter* **27**, 194110 (2015).

5. Pagni, R. M. & Compton, R. N. Asymmetric Synthesis of Optically Active Sodium Chlorate and Bromate Crystals. *Cryst Growth Des* **2**, 249–253 (2002).

6. Ismagilov, R. F., Schwartz, A., Bowden, N. & Whitesides, G. M. Autonomous Movement and Self-Assembly. *Angew. Chem. Int. Edit.* **41**, 652–654 (2002).

7. J. R. Howse *et al.*, Self-motile colloidal particles: From directed propulsion to random walk. *Phys. Rev. Lett.* **99**, 048102 , (2007).

8. P. Tierno, R. Golestanian, I. Pagonabarraga, F. Sagues, Controlled Swimming in Confined Fluids of Magnetically Actuated Colloidal Rotors. *Phys. Rev. Lett*. **101,** 218304 (2008),

9. A. Ghosh, P. Fischer, Controlled Propulsion of Artificial Magnetic Nanostructured Propellers. *Nano Lett.* **9**, 2243–2245 (2009).

10. S. Sanchez, A. N. Ananth, V. M. Fomin, M. Viehrig, O. G. Schmidt, Superfast Motion of Catalytic Microjet Engines at Physiological Temperature. *J. Am. Chem. Soc.* **133**, 14860–14863 (2011).

11. S. Thutupalli, R. Seemann, S. Herminghaus, Swarming behavior of simple model squirmers. *New J Phys*. **13**, 073021 (2011).

12. Y. Ikezoe, G. Washino, T. Uemura, S. Kitagawa, H. Matsui, Autonomous motors of a metal-organic framework powered by reorganization of self-assembled peptides at interfaces. *Nature Mat.* **11**, 1081–1085 (2012).

13. N. J. Suematsu, T. Sasaki, S. Nakata, H. Kitahata, Quantitative Estimation of the Parameters for Self-Motion Driven by Difference in Surface Tension. *Langmuir*. **30**, 8101–8108 (2014).

14. Kim, H., Muller, K., Shardt, O. *et al.* Solutal Marangoni flows of miscible liquids drive transport without surface contamination. *Nature Phys* **13,** 1105–1110 (2017)

15. Li Chen, Mao-Jie Zhang, Shi-Yuan Zhang, Lu Shi, Yi-Min Yang, Zhuang Liu, Xiao-Jie Ju, Rui Xie, Wei Wang, and Liang-Yin Chu *ACS Applied Materials & Interfaces* **2020** *12* (31), 35120-35131

16. Lee, E., Huang, D. & Luo, T. Ballistic supercavitating nanoparticles driven by single Gaussian beam optical pushing and pulling forces. *Nat Commun* **11,** 2404 (2020). https://doi.org/10.1038/s41467-020-16267-9
