# Supplementary material for: Unidirectional rotation of micromotors on water powered by pH-controlled disassembly of chiral molecular crystals
Source: Nat Commun. 2023 May 19;14:2869. doi: 10.1038/s41467-023-38308-9 (PMC10198998; doi:10.1038/s41467-023-38308-9)
Supplement: Supplementary file 3 — Description of Additional Supplementary Files [file 41467_2023_38308_MOESM3_ESM.pdf]

Description of Additional Supplementary Files

File Name: Supplementary Movie 1
Description: Chimot +

File Name: Supplementary Movie 2
Description: Chimot –

File Name: Supplementary Movie3
Description: 7 vessels chimot(-)

File Name: Supplementary Movie 4
Description: 7vessel chimot(+)

File Name: Supplementary Movie 5
Description: Chimot(+) with load

File Name: Supplementary Movie 6
Description: Chimot(-) with load

File Name: Supplementary Movie7
Description: Chimot(+) with NBH_4

File Name: Supplementary Movie 8
Description: Chimot(-) with NBH_4
